# Novel Viral Sequences in a Patient with Cryptogenic Liver Cirrhosis Revealed by Serum Virome Sequencing

**DOI:** 10.3390/v17060812

**Published:** 2025-06-03

**Authors:** Xiaoan Zhang, Ida X. Fan, Yanjuan Xu, Jody Rule, Long Ping Victor Tse, Mahmoud Reza Pourkarim, William M. Lee, Adrian M. Di Bisceglie, Xiaofeng Fan

**Affiliations:** 1 Division of Gastroenterology & Hepatology, Department of Internal Medicine, Saint Louis University School of Medicine, St. Louis, MO 63104, USA; zxa@vip.163.com (X.Z.); yanjuan.xu@health.slu.edu (Y.X.); adrian.dibisceglie@health.slu.edu (A.M.D.B.); 2 The Third Affiliated Hospital of Zhengzhou University, Zhengzhou 450001, China; 3 Department of Microbiology & Immunology, Saint Louis University School of Medicine, St. Louis, MO 63104, USA; ixf1@williams.edu (I.X.F.); victor.tse@health.slu.edu (L.P.V.T.); 4 Division of Digestive and Liver Diseases, Department of Internal Medicine, University of Texas Southwestern Medical Center, Dallas, TX 75390, USA; jody.rule@utsouthwestern.edu (J.R.); william.lee@utsouthwestern.edu (W.M.L.); 5 Laboratory for Clinical and Epidemiological Virology, Rega Institute, Department of Microbiology, Immunology and Transplantation, KU Leuven, 3000 Leuven, Belgium; mahmoudreza.pourkarim@kuleuven.be; 6 Saint Louis University Liver Center, Saint Louis University School of Medicine, St. Louis, MO 63104, USA

**Keywords:** etiology, hepatitis virus, cryptogenic liver disease, cryptogenic cirrhosis, virome

## Abstract

Clinical studies indicate the etiology of liver disease to be unknown in 5% to 30% of patients. A long-standing hypothesis is the existence of unknown viruses beyond hepatitis A through E virus. We conducted serum virome sequencing in nine patients with cryptogenic liver disease and identified eight contigs that could not be annotated. One was determined to be a contaminant, while two of seven contigs from an individual (Patient 3) were validated by reverse transcription and polymerase chain reaction (RT-PCR) and Sanger sequencing. The possibility of contamination was completely excluded through PCR, with templates extracted using different methods from samples taken at different time points. One of the contigs, Seq260, was characterized as negative-sense single-stranded DNA via enzymatic digestion and genome walking. Digital-droplet PCR revealed the copy number of Seq260 to be low: 343 copies/mL. Seq260-based nested PCR screening was negative in 200 blood donors and 225 patients with liver disease with/without known etiologies. None of the seven contigs from Patient 3 was mapped onto 118,713 viral metagenomic data. Conclusively, we discovered seven unknown contigs from a patient with cryptogenic liver cirrhosis. These sequences are likely from a novel human virus with a negative-sense, linear single-stranded DNA genome.

## 1. Introduction

Understanding the etiology of human disease is essential for effective prevention, diagnosis, and treatment. Many cases of liver disease have a clear etiology, for example, hepatitis virus infection, genetic abnormality, and immunological or metabolic disorder [1]. However, the underlying cause is reportedly unknown in 5% to 30% of patients with various types of liver disease, including hepatitis [2], cirrhosis [3], hepatocellular carcinoma [4], and acute liver failure [5]. A retrospective study of 135,191 cases reported that about 7% of patients who underwent liver transplantation were “cryptogenic”, that is, without known etiology [6]. These observations refresh a long-standing postulation for the existence of unknown hepatotropic viruses beyond hepatitis A through E viruses. Ongoing efforts to identify new hepatitis viruses have resulted in the discovery of multiple previously unknown viruses such as the anelloviruses [7], which include SEN virus [8], human pegivirus type 1 (HPgV-1, formerly called GB virus C or hepatitis G virus) [9,10], human pegivirus type 2 (HPgV-2; also known as human hepegivirus 1, HHpgV-1) [11,12], and the human circular double-stranded DNA (dsDNA) virus KIs_V [13]. Causal links with liver disease or other human diseases have not been established for any of these viruses, which are now considered commensal viruses in the frame of the human virome [14]. However, the sequential discovery of these diverse viruses implies a considerable abundance of unknown viruses within the human virome, which is largely unexplored [15]. The present study reports the discovery of novel virus-like sequences in a patient with cryptogenic liver cirrhosis.

## 2. Materials and Methods

### 2.1. Study Population and Patient Samples

We used two patient cohorts: one for viral discovery and one for screening. The discovery cohort consisted of nine patients with cryptogenic liver disease (Table 1). These patients had been excluded from the infection of known hepatitis viruses by routine laboratory tests. Serum samples were collected from these patients and archived in the Saint Louis University Liver Center Sample Repository. Written informed consent was obtained from each patient prior to sample collection; no treatment was given prior to sample collection. As a sole patient with positive discovery in the current study, Patient 3 (Table 1) visited the Saint Louis University Hospital in the middle of 1994 with a complaint of abdominal discomfort. Physical examination, lab tests, and imaging did not reveal obvious abnormality, except for a moderately elevated serum alanine aminotransferase (107 U/mL). Thus, the diagnosis of cryptogenic hepatitis was made. The patient attended regular follow-up visits, but did not receive any medical interventions and experienced raid disease progression, reaching the stage of cryptogenic cirrhosis by the end of 1995. Liver transplantation was carried out in early 1996, but the patient died of liver complications 6 months later. There was no recorded history of drug use, alcohol consumption, or previous surgery.

Samples from the screening cohort were categorized into three groups (Table 1): First, serum samples from patients with known etiologies, including hepatitis C virus (HCV) infection (n = 100) and hepatitis B virus (HBV) infection (n = 10), were accessed from our Liver Center Sample Repository. Second, serum samples from 200 blood donors were obtained from American Red Cross-National Testing Laboratory in Saint Louis as a gift to support biomedical research. The third group comprised samples from the National Institute of Diabetes and Digestive and Kidney Diseases Central Repository (NIDDK-CR), which archives patient specimens from NIDDK-sponsored clinical studies. We obtained serum samples from three completed clinical trials: the acute liver failure study group (ALFSG) (n = 50), the adult-to-adult living-donor liver transplantation cohort study (A2ALL) (n = 40), and the nonalcoholic fatty liver disease (NAFLD) adult (n = 24) (request numbers #23456, #23105, and #23744, respectively). The ALFSG samples were from patients who lacked known etiologies [5]. The A2ALL is a multi-center clinical trial that was conducted between 1998 and 2003 and aimed to determine whether adult-to-adult living-donor liver transplant has significant benefits for patients compared with deceased-donor liver transplantation [16]. All samples that we obtained from the A2ALL were collected from patients without explicit etiologies. The NAFLD adult samples were from patients with diagnosed cryptogenic cirrhosis at the time of recruitment [17]. All serum samples from the NIDDK-CR were coded prior to shipment to our lab. The entire research protocol for the use of patient samples was reviewed and approved by the Saint Louis University Institutional Review Board (IRB protocol: SLU10592).

### 2.2. Serum Virome Sequencing

Serum virome sequencing was performed as previously described [18,19]. Briefly, total RNA was extracted from 140 μL of serum and eluted into 60 μL Tris buffer (pH 8.5) using the QIAamp Viral RNA Mini kit (Qiagen, Germantown, MD, USA). According to the manufacturer’s instructions, the kit extracts both DNA and RNA larger than 200 bp. For reverse transcription (RT), 10.6 μL of extracted RNA was mixed with 9.4 μL RT matrix consisting of 1× SuperScript III buffer, 10 mM dithiothreitol (DTT), 80 μM of primer C28 (Table 2), exonuclease-resistant random pentamer primers with the 5′ end blocked by C18 spacer [20], 1 mM dNTPs (Life Technologies, Carlsbad, CA, USA), 20 U of RNasin ribonuclease inhibitor (Promega, Madison, WI, USA), and 200 U of SuperScript III reverse transcriptase (Life Technologies). The reaction was incubated at 37 °C for 30 min, 50 °C for 30 min, and inactivated by incubation at 70 °C for 15 min. A 4 μL aliquot of RT was used for template-dependent Multiple Displacement Amplification (tMDA) in a 40 μL reaction volume, consisting of 1× phi29 DNA polymerase buffer, 1 mM dNTPs, 80 μM primer C28 (as used in the RT), and 20 units of phi29 DNA polymerase (New England Biolabs, Ipswich, MA, USA). The reaction was incubated at 28 °C for 14 h and then terminated by heating at 65 °C for 15 min. After purification using the QIAamp DNA mini kit (Qiagen, Germantown, MD, USA), the product of RT-tMDA was subjected to library construction with the Nextera XT DNA Sample Preparation kit (Illumina, San Diego, CA, USA), followed by sequencing on the Illumina NextSeq 500 platform (1× 250-bp single-end reads, mid-output mode) at MOgene (St. Louis, MO, USA).

### 2.3. Viral Categorization and Discovery

Raw sequence reads in FASTQ format from each sample were filtered using PRINSEQ (v0.20) for quality control with the following parameters: read length ≥70 nt, mean read quality score ≥25, low complexity with DUST score ≤7, ambiguous bases ≤1%, and all types of duplicates [21]. We used Bowtie 2 mapper (version 2.5.3) to map quality reads onto viral reference sequences from the National Center for Biotechnology Information (NCBI) (18,677 complete viral genomes downloaded in March 2025) [22,23]. After viral mapping, reads were filtered by subtractive mapping from the two non-template controls; human sequences (GRCh38 build); NCBI microbial reference sequences for bacteria, archaea, fungi, and protist (downloaded in March 2025) [23]; and microbial reference genomes from the Human Microbiome Project [24]. The remaining reads were de novo assembled using SPAdes (version 3.13.0), a short-read assembler [25]. Assembled contigs were labeled in PRINSEQ and combined to generate a new dataset, which was then filtered in CD-HIT based on 90% nucleotide similarity [26], followed by similarity-based annotation using NCBI BLASTN against the NCBI collection of nucleotide acid sequences (database “nt”) with a conserved E-value setting of 1 × 10^−5^. Contigs with no BLASTN hits were translated in all six reading frames and searched using BLASTP against the NCBI nonredundant protein database (“nr”) with the E-value setting of 1 × 10^−5^. Contigs without BLASTP hits were evaluated for remote homology by Profile Hidden Markov Model (HMM) analysis in HMMER (v3.2.1) [27] with the HMM-profiles built from NCBI viral RefSeq except for phage (vFam) [28], phage [29], and the collection of protein families (Pfam) (24,076 entries in version 37.2) [30]. Final contigs without any hits throughout all analyses were considered to be unknown sequences.

### 2.4. Validation of Unknown Sequences

To exclude the possibility of contamination from reagents or experimental pipelines, three unknown sequences were selected for validation by RT-PCR directly from corresponding serum. Briefly, 10 μL of total RNA was used for RT in a 20 μL reaction volume, as described above, except that sequence-specific primer R1 was used (Table 2). A 5 μL aliquot of the RT product was used for the first round of PCR in a 50 μL reaction including 1× Q5 polymerase buffer, 0.8 mM dNTPs, 0.4 μM each of primers R1 and F1 (Table 2), and 1 U of Q5 DNA polymerase (New England Biolabs, Ipswich, MA, USA). Cycle parameters were programmed as 94 °C for 2 min, connected by the first 5 cycles of 94 °C for 1 min, 60 °C for 1 min, and 72 °C for 1 min, linked by 25 cycles in which the annealing temperature was reduced to 50 °C (referred to as the “touchdown protocol”), followed by a final 7-min incubation at 72 °C. A 2 μL aliquot of the first-round PCR product was used for the second round of PCR using the same cycle parameters and primers F2 and R2 (Table 2). The product was gel-purified and subjected to Sanger sequencing. After validation, PCR was repeated with and without RT using freshly prepared templates, including total serum RNA re-extracted using the QIAamp Viral RNA Mini kit (Qiagen, Germantown, MD, USA), Qiagen column-flushed water, and total DNA extracted with the Apostle MiniMax High Efficiency Cell-Free DNA Isolation Kit (Apostle, Pleasanton, CA, USA).

### 2.5. Machine-Learning Analysis for the Origin of Unknown Sequences

All unknown sequences were evaluated for the likelihood of viral origin using VirFinder, based on the different *k*-mer signatures of viral and bacterial genomes [31]. Analyses were conducted under two models: Firstly, the VF.modEPV_k8.rda model implemented in VirFinder was used to distinguish bacteria and/or archaea from prokaryotic and/or eukaryotic viruses. This model was trained with 5800 viral genomes from the NCBI viral RefSeq [31]. The second model was trained to separate 142,809 newly assembled human phage genomes from 2206 complete genomes of known hepatitis viruses from GenBank, comprising 73, 1054, 1040, 7, and 32 genomes from hepatitis A, B, C, D, and E viruses, respectively. Based on the trained models, the program extracted *k*-mer features from query sequences to generate scores ranging from 0 to 1, with 1 representing the highest likelihood of a viral sequence. Scores were compared with the distribution of scores from the training sequences to compute statistical significance. Contigs with a low score and high *p*-value (>0.05) were considered unlikely to be of viral origin.

### 2.6. Genome Walking and Strand Attribute Determination

We selected one unknown sequence, namely Seq260, for both experiments. Genome walking consisted of four steps: primer extension (PE), intramolecular circularization, rolling-cycle amplification (RCA), and PCR and Sanger sequencing. Briefly, for PE at the 5′ end, 5 µL of extracted DNA was used in a 50 µL reaction consisting of 0.4 µM primer PAWR (Table 2), 1× Q5 reaction buffer, and 1.6 U Q5 DNA polymerase. After incubation at 94 °C for 2 min, the mixture was subjected to 30 cycles of the touchdown protocol described above. The PE product was purified into 10 µL of elution buffer using MinElute PCR Purification Kit (Qiagen, Germantown, MD, USA), and 8 µL of the purified PE product was used for intramolecular ligation in a 20 µL reaction finalized with 1× CircLigase II buffer, 2.5 mM MnCl_2_, 1 M Betaine, and 150 U CircLigase II ssDNA Ligase (Lucigen, Middleton, WI, USA). The reaction was incubated at 60 °C for 3 h, then inactivated at 80 °C for 10 min, and finally purified using the MinElute PCR Purification Kit (Qiagen, Germantown, MD, USA). The total purified ligation product (10 µL) was used for RCA with 20 U phi29 DNA polymerase (New England Biolabs, Ipswich, MA, USA) and 0.4 µM each of primers AWF7bp and AWR7bp (Table 2). These 7 nt primers were selected from all 7 nt strings of the PE region of Seq260 by primer PAWR with 1 nt overlap based on GC content, melting temperature, and the number of perfect replicates in the human reference genome (GRCh Build 38), as described previously [32]. The use of 7 nt but not 6 nt primers in RCA aimed to reduce the number of their replicates on the human genome. After incubation at 30 °C for 16 h, the RCA reaction was inactivated at 65 °C for 10 min, and then it was purified into 30 µL of elution buffer, using the QIAamp DNA Mini Kit (Qiagen, Germantown, MD, USA). A 5 µL aliquot of purified RCA product was subjected to 60 cycles of nested PCR as described above, except that primers AWF1 and AWR1 were used in the first round of PCR, and primers AWF2 and AWR2 were used in the second round of PCR (Table 2). The resulting PCR product was gel-purified and used for Sanger sequencing. The entire procedure was repeated to perform genome walking at the 3′ end with the use of different primers (Table 2). The RCA step in genome walking at the 3′ end used primers BWF7bp and AWR7bp. To evaluate whether Seq260 was single- or double-stranded, 12 µL of extracted DNA was digested with 20 U of the type II restriction enzyme NruI (New England Biolabs, Ipswich, MA, USA) in a 20 µL reaction volume at 37 °C for 1 h. A 3 µL aliquot of this reaction without inactivation was directly used as the input of nested PCR, after which the product was visualized on agarose gel. The same procedures were applied to the total DNA extracted from a mock serum sample consisting of 1 × 10^6^ copies/mL synthetic double-stranded gBlocks Seq260 (Integrated DNA Technologies, Coralville, IA, USA).

### 2.7. Determination of Copy Numbers of Seq260 Using Digital Droplet PCR

The digital-droplet PCR (ddPCR) mixture was prepared in a semi-skirted 96-well plate (Bio-Rad, Hercules, CA, USA). Each well contained 20 µL reaction volume, including 1× ddPCR supermix for probes (no dUTP), 0.5 µM each of primers 260F and 260R, 0.25 µM of probe 260P (Table 2), and the DNA inputs. After the sealing with a pierceable foil heat seal (Bio-Rad, Hercules, CA, USA) at 180 °C for 5 s, the plate was applied to a Bio-Rad QX200 Automated Droplet Generator (Bio-Rad, Hercules, CA, USA) to generate at least 10,000 droplets. Droplets were transferred to a Bio-Rad PX1 PCR Plate, and standard PCR was run in the Bio-Rad C1000 Thermal Cycler (Bio-Rad, Hercules, CA, USA) with the following program: 10 min at 95 °C for enzyme activation, followed by 40 cycles of 94 °C for 30 s and 60 °C for 1 min, and then deactivation at 98 °C for 10 min. After PCR, the plate was submitted to Bio-Rad QX200 Droplet Reader (Bio-Rad, Hercules, CA, USA), and data were analyzed using Bio-Rad QX Manager 1.2 software (Bio-Rad, Hercules, CA, USA). The input in the ddPCR included an aliquot of 2.5 ng of extracted DNA from the Seq260-positive sample, 10 fg gBlocks Seq260 used as the positive control, and 2.5 ng each of extracted DNA from the mock serum samples, prepared in a 1:10 serial dilution to contain different copy numbers of gBlocks Seq260 in the serum from a healthy blood donor.

### 2.8. Detection of Seq260 in the Screened Cohort

Total DNA was extracted from serum samples in the screened cohort using Apostle MiniMax High Efficiency Cell-Free DNA Isolation Kit (Apostle, Pleasanton, CA, USA). An aliquot of 2.5 ng of extracted DNA was used as the input for the nested PCR with primers designed based on Seq260 (Table 2). The product of the PCR was visualized on agarose gel. Suspected amplicons were gel-purified and subjected to Sanger sequencing.

### 2.9. In Silico Screening of Unknown Sequences

All unknown sequences were examined for possible existence in previously published next-generation sequencing (NGS) data. First, we searched the National Center for Biotechnology Information sequence read archive (SRA) portal using the key words “virome”, “viral metagenomics”, “serum”, or “plasma”. Available NGS datasets were downloaded and directly used for mapping with gapped mapper Bowtie 2 or BWA [33]. Second, we screened whole-genome sequencing (WGS) data from the BioMe Biobank at Mount Sinai, which is a community cohort study with 10,178 participants in the National Heart, Lung, and Blood Institute (NHLBI) Trans-Omics for Precision Medicine (TOPMed) initiative [34]. These data are archived in the Cloud as compressed reference-oriented alignment maps (CRAMs) and were analyzed with a memory-focused instance type r5a.12x (48 vCPU and 384 GB memory) in Amazon Web Services. After downloading with the NCBI SRA Toolkit (version 3.1.0) (option “type all” for CRAM format) [35], data were processed in SAMTools to extract unmapped reads (using the option “f4”) [36], followed by mapping with Bowtie 2. The DIAMOND BLASTX is a faster algorithm than NCBI BLASTX, which we applied for additional analyses of unmapped reads at the amino acid level [37]. Finally, we screened non-human reads of metagenomics and metatranscriptomics data from nine children with acute hepatitis of unknown etiology [38] (kindly provided by Sarah Buddle, Dr. Sofia Morfopoulou, and Professor Judith Breuer at University College London). The in silico screening protocol for unknown sequences was independently reviewed and approved by the Saint Louis University Institutional Review Board (IRB protocol: SLU34248).

## 3. Results

### 3.1. Discovery of Novel Virus-like Sequences in a Patient with Cryptogenic Liver Cirrhosis

**Figure 1:**
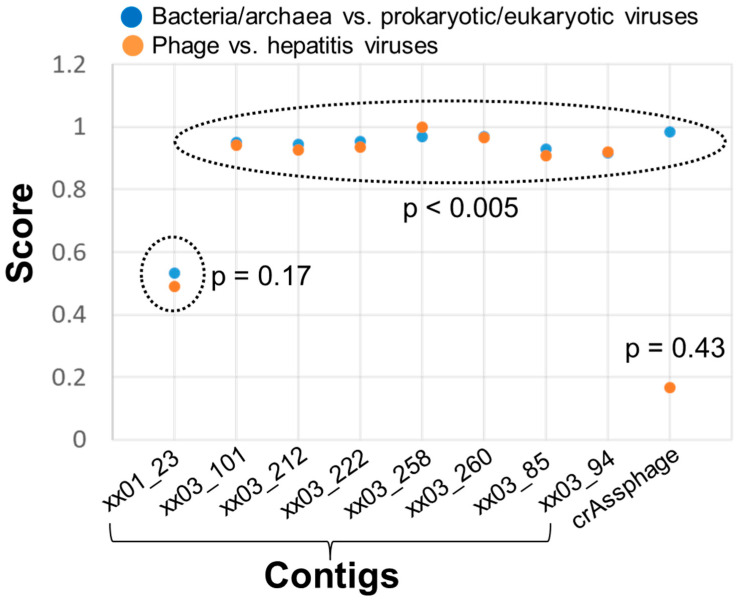
A machine-learning analysis of eight unknown contigs as viral sequences. The crAssphage genome (GenBank accession number NC_024711) was used as input to evaluate the performance of the two trained models applied in analyses. Eight unknown contigs were found to have very similar *p*-values with both models so that only a single *p*-value was shown for contig xx01_23 from Patient 1. The *p*-values of the seven unknown contigs from Patient 3 were all ≤ 0.005.

Serum virome sequencing generated an average of 4.89 ± 0.34 million reads per case. Viral categorization revealed the existence of known human viruses, including anelloviruses and phages. As expected, no known hepatitis viruses were detected in any of the nine patients in the discovery cohort. A total of 28,243 reads remained after subtractive mapping and which were assembled into 5370 contigs. Subsequent annotation identified unknown sequences of only eight contigs: one from Patient 1, and the other seven from Patient 3 (Supplementary Table S1). These eight contigs had no hits in any similarity-based searches at either the nucleotide or amino acid level. Machine-learning analysis revealed contig xx01_23 from Patient 1 to have a score of around 0.5 in both models, and such a score is not indicative of viral origin. However, all seven contigs from Patient 3 had scores above 0.9, regardless of the model applied (Figure 1). Interestingly, the score of crAssphage dropped significantly in the phage/hepatitis virus model, suggesting that *k*-mer frequency differs between phage and hepatitis virus genomes.

### 3.2. Seq260 (Contig xx03_260) from Patient 3 Is Not a Contaminant

**Figure 2:**
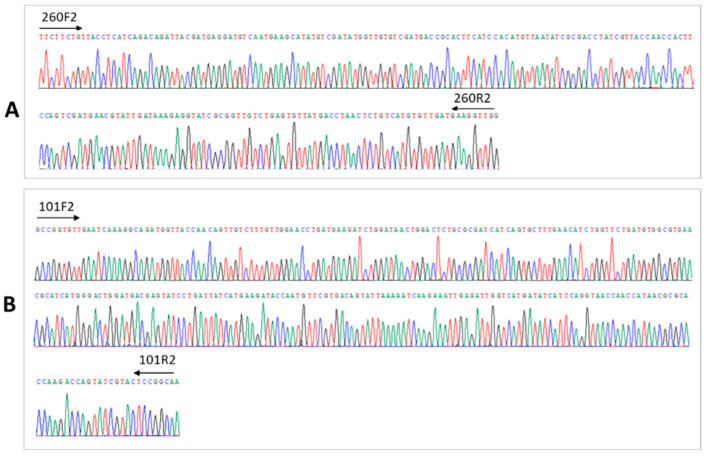
Results of Sanger sequencing of the amplicons from contigs xx03_260 (**A**) and xx03_101 (**B**). Each amplicon was sequenced in both directions. Priming sites for sequencing are indicated.

**Figure 3:**
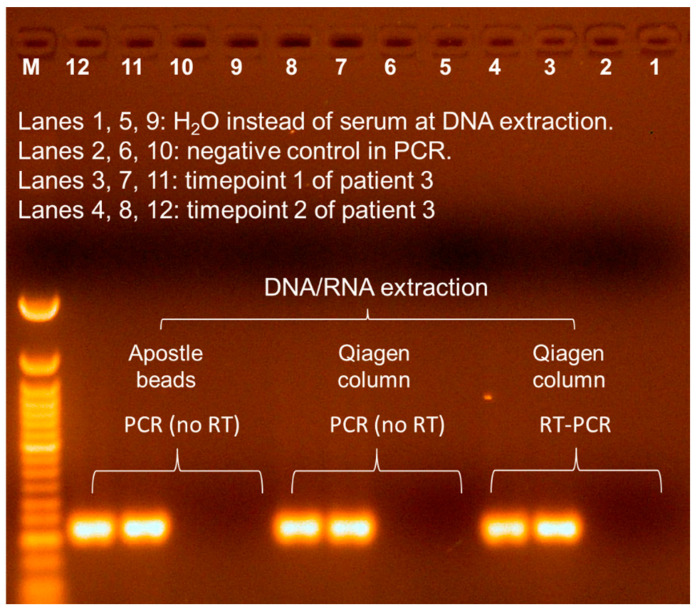
Summarized (RT) PCR results for samples from Patient 3. Serum samples were collected from Patient 3 at two timepoints. M, 50 bp DNA ladder (NEB).

Three contigs (xx01_23 from Patient 1; and xx03_101 and xx03_260 from Patient 3) were selected for validation from the corresponding serum samples. No amplicon was detected in contig xx01_23-based RT-PCR, despite repeated experiment. The two contigs from Patient 3 were validated using RT-PCR on extracted nucleic acids from this patient. Sanger sequencing of the gel-purified PCR product revealed 100% identity to the assembled contigs (Figure 2). Contig xx03_260 was denoted Seq260 and used in subsequent experiments. No amplification was achieved by repeating Seq260-based RT-PCR using Qiagen column-flushed water (Figure 3). Furthermore, a product of the same size was detected following PCR without RT, suggesting that Seq260 is a DNA rather than an RNA sequence (Figure 3). We also detected Seq260 in total serum DNA extracted using Apostle MiniMax High Efficiency Cell-Free DNA Isolation Kit (Figure 3). The Apostle kit that we used is a bead-based serum DNA extraction kit, free of any kind of silica (Fan and Apostle, communication). Finally, there were two serum samples from Patient 3 that were respectively collected at two separate time points prior to liver transplantation. Seq260 was positive in both serum samples (Figure 3).

### 3.3. A Net Extension of 107 nt at the 3′ End of Seq260 by Genome Walking

**Figure 4:**
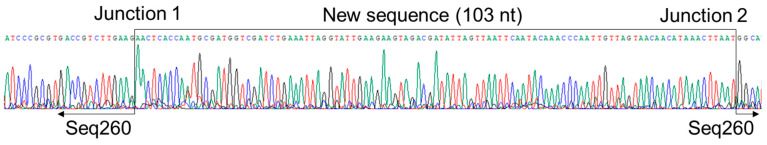
Results of genome walking at the 3′ end of Seq260. The Sanger sequencing map is shown for the domain covering the novel 103 nt sequence that was extended with genome walking. Two junction sites between the known Seq260 and newly extended sequences are indicated.

**Figure 5:**
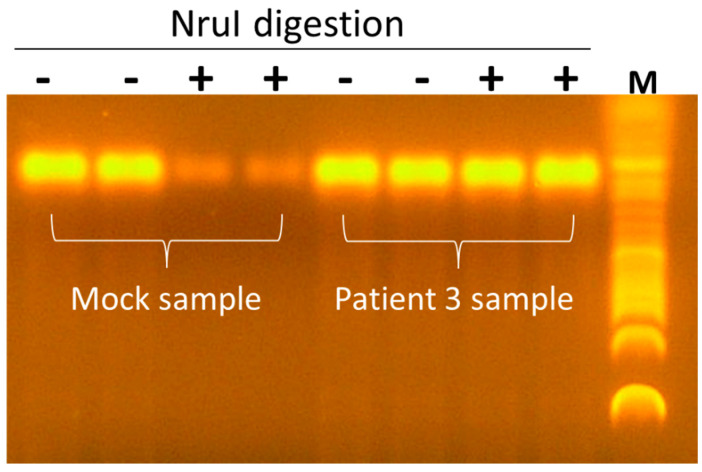
PCR amplification of the putative viral genome with and without NruI digestion.

Genome walking at the 3′ end of Seq260 resulted in the detection of an amplicon with a size of ~220 bp. Sanger sequencing confirmed the success with a net extension of 103 nt at the 3′ end (Figure 4). However, genome walking at the 5′ end of Seq260 failed despite repeated experiment. An additional 103 nt from genome walking at the 3′ end gives Seq260 490 nt in length; however, the sequence remains unable to be annotated. In addition, PCR using the NruI-digested template (the recognition site is in the middle of Seq260) exhibited no signs of reduced PCR amplification efficiency compared with the mock sample containing synthetic double-stranded gBlocks Seq260 (Figure 5).

### 3.4. Seq260 Is Not Detected in Other Patients and Next-Generation Sequencing Data

**Figure 6:**
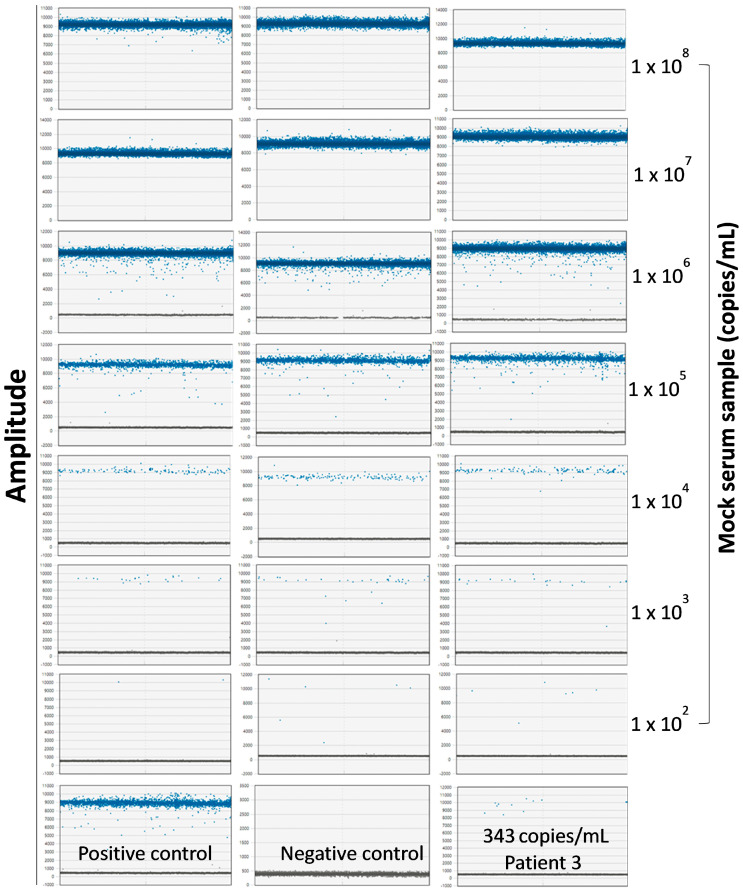
Quantitation of Seq260 copy number in serum. Amplitude outputs of digital-droplet PCR (ddPCR) from Patient 3 (bottom right) and the mock serum samples with concentrations of gBlocks Seq260 from 100 to 1 × 10^8^ copies/mL. Each mock serum sample was set up with three technical replicates.

The readouts of Seq260-based ddPCR became saturated in the mock serum samples with concentrations ≥ 1 × 10^7^ copies/mL, as indicated by the lack of negative droplets (Supplementary Figure S1). We evaluated our ddPCR to have a sensitivity of 100 copies/mL (Figure 6). However, reproducible quantitation, shown as comparable numbers of positive droplets among three technical replicates, was observed in the mock samples with a concentration of no less than 1 × 10^3^ copies/mL (Figure 6). Seq260 was found to be present at a concentration of 343 copies/mL in serum from Patient 3 (Figure 6), a low titer that necessitated the use of nested PCR to enable visualization of the PCR product on agarose gel. Despite the use of nested PCR, Seq260 was not detected in a total of 433 serum samples from both cohorts and was only detected in samples from Patient 3. Amplicons larger than the expected size of 237 bp were observed in four of the 433 (0.9%) serum samples. However, Sanger sequencing revealed these to be human sequences, indicating non-specific amplification. For in silico screen, keyword searches returned NGS data from 118,731 bio-samples in 617 bio-projects, about 78.3 TB in size. Of the total 118,731 bio-samples, 92,058 (77.5%) were from The Environmental Determinants of Diabetes in the Young (TEDDY) study [39]. Contig xx01_23 from Patient 1 was mapped with ≥ 1 read (s) in 14 bio-samples from five bio-projects (Supplementary Table S2). In contrast, none of seven contigs from Patient 3 had hits in any of the 118,731 NGS datasets. Non-human reads accounted for 1.82 ± 1.44% of the 10,178 WGS data in the TOPMed BioMe Biobank (185 TB in CRAM format), none of which was mapped onto the eight contigs in the present study. Diamond BLASTX analysis of the eight contigs also revealed no meaningful hits on these reads under the definition of the E-value ≤ 1 × 10^−5^. Finally, mapping and Diamond BLASTX analysis showed the lack of hits of all unknown sequences on non-human reads from samples in children with cryptogenic hepatitis.

## 4. Discussion

The advent of NGS technology has greatly advanced viral discovery. However, contamination is a major issue for NGS-based approaches [40], especially when working with low-biomass samples like patient serum. The presence of contaminants can lead to misinterpretation of the data [41]. The current study identified eight unknown contigs from virome sequencing data. We were unable to validate contig xx01_23 from Patient 1 directly from the serum sample. Machine-learning analysis indicated that this contig was not a viral sequence. In addition, the contig was detected in multiple NGS data associated with various clinical phenotypes, even in the tick virome [42,43,44,45]. Taken together, these findings lead us to conclude that contig xx01_23 is likely a contaminant. In contrast, we have obtained multiple lines of evidence to suggest at least two of seven unknown contigs, namely xx03_260 (Seq260) and xx03_101, from Patient 3 are authentic sequences. Direct detection of Seq260 in serum samples obtained at two time points indicates that Seq260 is unlikely to be a contaminant from phlebotomy, reagents, or any steps of the experimental protocol. Furthermore, the strong support from machine-learning analysis indicates that these seven unknown contigs are viral in origin, likely from a single unknown human eukaryotic DNA virus.

The success of genome walking at the 3′ but not the 5′ end indicates that Seq260 may have only one strand. All primers in genome walking were designed based on a positive-sense orientation of Seq260 in accordance with the coding direction of its largest open reading frame (ORF) of 127 amino acids. Successful genome walking at 3′ end but not 5′ end suggests a negative-sense orientation of Seq260. In addition, most type II restriction enzymes preferentially cleave dsDNA over single-stranded DNA (ssDNA) [46]. Digestion with type II restriction enzyme NruI targeting the middle of the Seq260 did not reduce the amplification signal, further confirming the sequence to be a single-stranded DNA. Finally, all seven unknown contigs from Patient 3 were assembled from 42 of 4,101,707 (0.001%) reads from the RT-tMDA product. The phi29 DNA polymerase used in tMDA is well known to favor a circular over a linear DNA template [47]. For example, human anelloviruses are ubiquitous commensal viruses with circular ssDNA genomes, present in serum at usually very low loads (about 1 × 10^2^ to 1 × 10^3^ copies/mL) [48]. Virome sequencing using RT-tMDA can readily detect these viruses, which account for an average of ≥5% reads, in serum samples from patients and blood donors [18,19]. Thus, extremely low readouts in NGS suggest that the putative virus in Patient 3 is likely to have a negative-sense linear ssDNA genome.

This putative virus was not detected in the screening cohort or in archived NGS data relevant to the host virome studies. Notably, our screening cohort included patients with different liver disorders of unknown etiologies. Similarly, in silico screening covered NGS data of patients with cryptogenic liver disease, such as acute liver failure (SRA accession number: PRJNA389455) [49], post-transfusion hepatitis (SRA accession number: PRJNA217527) [41], and unexplained acute hepatitis in children [38]. However, a negative detection in experimental and in silico screening does not necessarily mean that the putative virus is not present in these subjects for a number of reasons: First, the proportion of patients with liver disease and no clear etiology is small, but the absolute number of patients with cryptogenic liver disease is large due to a big patient reservoir. Thus, the screening cohort in the present study is relatively small. Second, screening PCR was based on Seq260, which appears to be a coding domain. Mutations in coding domains are common in ssDNA virus, such as in the case of human anelloviruses, where PCR based on a 500 nt coding DNA fragment that was initially discovered indicated the prevalence of these viruses to be 3.3%. However, when PCR was carried out targeting the non-coding region, the viruses were found to be ubiquitous in the human population [50]. Finally, the very small size of most human viral genomes results in overwhelming domination of host nucleic acids in human samples in terms of molecular weight, leading to extremely low viral output in NGS-based virome sequencing data and remaining an unsolved technical issue [51]. For this reason, complete genome sequencing of severe acute respiratory syndrome coronavirus 2 (SARS-CoV-2) using NGS is recommended by the World Health Organization (WHO) only in cases where the Ct values in real-time PCR is below 30 [52]. A well-known human linear ssDNA virus is parvovirus B19, which can package either the negative or positive DNA strands of its 5.6 kb genome into viral particles [53]. By analogy, it is plausible that our putative virus has a similar genome size. The small genome and potentially low viral load, as seen in Patient 3, could explain the negative result of in silico screening of virome sequencing data. Taking together, a cautious conclusion from our data is that the putative virus has a very low population frequency.

The present study has some limitations which should be acknowledged. The total volume of serum samples available at two time points from Patient 3 was only 0.7 mL. The limited volume did not allow for further experimentation to characterize the putative virus. In addition, only serum samples were available from patients with cryptogenic liver disease, including Patient 3. This excluded the possibility for investigation of the putative virus directly in liver tissue. We are, therefore, unable to draw definitive conclusions with regard to etiological associations between the putative virus and cryptogenic liver disease. However, the data presented here lead us to conclude with confidence that we have discovered seven unknown sequences in a serum sample from a patient with cryptogenic liver cirrhosis. These unknown sequences are likely from a novel human virus with a negative-sense linear ssDNA genome. Further work is anticipated to conduct an extended screening in general and target populations. Once additional subjects carrying the putative virus are identified, it will be feasible to determine the complete genome sequence, allowing comprehensive genome annotation and downstream experimentation, such as serological studies. Finally, our study also signals a challenge in investigating unannotated sequences from viral metagenomics data since it is likely a mixture of contaminants and real viral elements. The methods presented in the current study should be helpful for the development of generalized pipelines to unveil the nature of this so-called viral dark matter [15].

## Figures and Tables

**Table 1:** The list of patients used in the current study. Note that the diagnosis in the cohort for viral discovery is based on the record of patient’s last visit at Saint Louis University Hospital. Asterisk indicates that these patients have unknown etiologies from the parent studies. Abbreviations: CLD, cryptogenic liver disease; OLT, orthotopic liver transplantation; SLULC, Saint Louis University Liver Center; ARC, American Red Cross; NIDDK-CR, the National Institute of Diabetes and Digestive and Kidney Diseases Central Repository; NA, not available.

**Cohort for Viral Discovery**						
**Patient #**	**Sex**	**Age**	**Diagnosis (CLD)**	**Outcome**	**Specimen**	**Source**
1	F	64	Cirrhosis	NA	Serum	SLULC
2	F	73	Hepatitis	NA	Serum	
3	F	60	Cirrhosis	OLT, deceased	Serum	
4	F	42	Cirrhosis	NA	Serum	
5	F	68	Cirrhosis	deceased	Serum	
6	M	40	Cirrhosis	NA	Serum	
7	M	59	Cirrhosis	OLT, deceased	Serum	
8	M	65	Cirrhosis	NA	Serum	
9	M	54	Hepatitis	NA	Serum	
**Cohort for PCR Screening**						
**Group #**	**Diagnosis**			**Number**	**Specimen**	**Source**
1	Chronic HCV infection			100	Serum	SLULC
	Chronic HBV infection			10	Serum	
2	Blood donors			200	Serum	ARC
3	Acute liver failure			50 *	Serum	NIDDK- CR
	Liver transplantation			40 *	Serum	
	Cirrhosis			25 *	Serum	

**Table 2:** The list of primers used in the current study. All primers were designed according to the orientation of the contigs that could generate the largest open reading frame (ORF), starting with any sense codon in a positive-sense manner, as predicted in the NIH ORFfinder. Star donates phosphorothioate bonds in primers for the resistance to exonuclease activity of phi29 DNA polymerase. Primers PAWR and PBWF had their 5′ ends modified by phosphorylation. Primer C28 had its 5′ ends blocked by C18 spacer. Asterisk donated phosphorothioate bonds to resist exonuclease activity of phi29 DNA polymerase. All primers and probes were synthesized in the Integrated DNA Technologies (Newark, NJ). Abbreviation: NA, not applicable; FAM, 6-carboxytetramethylrhodamine; BHQ1, black hole quencher 1.

Application	Target		Primer Name	Polarity	Sequence (5′→3′)	Amplicon Size
RT-PCR/PCR Validation	Contig xx12_260 (Seq260)		F1	Forward	tccttgatgcaagccattg	237 bp
			R1	Reverse	gcgggataccaacaacaac	
			F2	Forward	atgtcactggcatccttcttc	
			R2	Reverse	taccaacaacaacccaacc	
	Contig xx12_101		F1	Forward	gatggtgtccccactacagc	305 bp
			R1	Reverse	acaactcacgaccaggaacc	
			F2	Forward	tttaagcagtggtatgccggt	
			R2	Reverse	accatgttggtaattgccgga	
	Contig xx01_23		F1	Forward	cgatcaagtactctcgccga	237 bp
			R1	Reverse	gccatcacatgcatcaggaa	
			F2	Forward	gtactctcgccgatacgtct	
			R2	Reverse	agcatcaaccgaaaagccag	
Genome walking	Seq260	5′ end	PAWR	Reverse	phos-catcgactggaagtggttgg	NA
			AWF1	Forward	gaccgcacttcatccacatg	
			AWR1	Reverse	atgcttcattgacatcctcatc	
			AWF2	Forward	cgcgacctatcgttaccaac	
			AWR2	Reverse	cagaagaaggatgccagtgac	
			AWF7bp	Forward	taatc*c*g	
			AWR7bp	Reverse	ggata*c*c	
		3′ end	PBWF	Forward	phos-ggcatccttcttctgttacctc	NA
			BWF1	Forward	cctatcgttaccaaccacttcc	
			BWR1	Reverse	atgaagtgcggtcatcgac	
			BWF2	Forward	aggtatcgcggttgtctgag	
			BWR2	Reverse	atgcttcattgacatcctcatc	
			BWF7bp	Forward	ctaac*t*c	
ddPCR	Seq260		260F	Forward	cagacagattacgatgaggatgt	100 bp
			260R	Reverse	ggtaacgataggtcgcgatatt	
			260P	Reverse	FAM-tcgatgaccgcacttcatccacat-BHQ1	
MDA			C28	NA	/5Sp18/nnn*n*n	20 kb

## Data Availability

Raw Illumina sequence data after the removal of human sequences are available upon request made to the corresponding author. A total of eight unannotated contigs are reported in the Supplementary Materials (Table S1). The two contigs validated in the current study were deposited in the GenBank under the accession numbers MW468091 and PV170683 for contigs xx03_260 (Seq260) and xx03_101, respectively.

## Supplementary Materials

The following supporting information can be downloaded at https://www.mdpi.com/article/10.3390/v17060812/s1, Table S1. Summary of eight contigs that cannot be annotated from virome analysis; Table S2. Screen of contig xx01_23 in virome sequencing data via read mapping.

## Abbreviations

RT-PCR, reverse transcription and polymerase chain reaction; ALF, acute liver failure; HCV, hepatitis C virus; HBV, hepatitis B virus; dsDNA, double-stranded DNA; NIDDK-CR, the National Institute of Diabetes and Digestive and Kidney Diseases Central Repository; ALFSG, acute liver failure study group; A2ALL, adult-to-adult living-donor liver transplantation cohort study; NAFLD, nonalcoholic fatty liver disease; tMDA, template-dependent multiple displacement amplification; NCBI, National Center for Biotechnology Information; HMM, Hidden Markov Model; PE, primer extension; RCA, rolling-cycle amplification; ddPCR, digital droplet PCR; NGS, next-generation sequencing; SRA, sequence read archive; WGS, whole-genome sequencing; NHLBI, National Heart, Lung, and Blood Institute; TOPMed, Trans-Omics for Precision Medicine; ssDNA, single-stranded DNA; WHO, World Health Organization; SARS-CoV-2, severe acute respiratory syndrome coronavirus 2.
