# Supplementary material for: Novel Viral Sequences in a Patient with Cryptogenic Liver Cirrhosis Revealed by Serum Virome Sequencing
Source: Viruses. 2025 Jun 3;17(6):812. doi: 10.3390/v17060812 (PMC12197384; doi:10.3390/v17060812)
Supplement: Supplementary file 1 [file viruses-17-00812-s001.zip › viruses-3676367-supplementary.pdf]

Title: Novel Viral Sequences in a Patient with Cryptogenic Liver Cirrhosis Revealed by Serum Virome Sequencing

Supporting information

Table S1. Summary of eight contigs that cannot be annotated from virome analysis.
Table S2. Screen of contig xx01_23 in virome sequencing data via read mapping

Patient	Contig ID	Length (nt)	Reads Supported	GC content (%)
1	xx01_23	493	7	0.6085
3	xx03_101 (Seq101)	736	11	0.4198
	xx03_212	435	2	0.4023
	xx03_222	428	4	0.4393
	xx03_258	390	2	0.3897
	xx03_260 (Seq260)	387	3	0.4315
	xx03_85	839	12	0.4577
	xx03_94	777	8	0.4234

```
>xx01_23
GGGTGTAGAGAATCACCCGCGGCGCCCGCGCCGAACCGATCTGCGCGCTCTTGCTGGCGGGGGCGATCAAGTACTCTCGCCGATACGTCTCCGGAAGGCAGCAAATTATGTCTTCGGGCTTTTGGA
TTTTCGCCTTCAGGCTCGGCGTCACTTCGCCGGTCACCGTGAGAGATTCGATCTCCGACTCCGTAAGCTCGCGGCCTAGGGGGCAGGACGCGGAAACGTCCGCGGAAGCCACCGGGGGCGTTGCCC
CGAAGAGCGCCGATATCGTGAGCCAAAGCACCATCCTGGCTTTTCGGTTGATGCTTGGGGAGGAATTAGGAGTTGCGGTTACCGTTACCAGGTAACGCGGCCCGCGATGAGCGAGGCTCGAAGCGG
CGATCGGCAAACTCCCTTGCCGGCATTTTTTTTTGTGAGTAACAAGTATTTCCTGATGCATGTGATGGCGTGGATCGGGATCGGCCGCCTTCGGCTCGCGGGGTGCGCAAAAGGC
>xx12_101
GCCCTTTAATCAAACTTTAATATAGAGAGAGAAATAAAATGGAACAACAAGATATCGTAGCAACTACTACACTACAGCTTCTTCTGAATGCACAGCGTAAATATAATCGCGAATTCTTTATGGTTG
TTGATGGTGTCCCCACTACAGCAATTCTGACTCACTTTAATGGTATCGTTAAACAACTGTTTGATATCGACGGTCCTACTCTATTACCTACTGACTCATTAACTCCAGTCAAGATGTTTAAGCAGT
GGTATGCCGGTGTTGAATCAAAGGCAGATGGTTACCAACAGTTGTCTTTGTTGGAACCTGATGAAGATCTGGATAACTGGACTCTGCGCGATCATCAGTGCTTTGAACATCTGGTTCTGATGTGGC
GTGAACGCATCATGGGACTGGATGACGAGTATCCTGATTATCATGAAGATACCAATGTTCGTGACAGTATTAAAAATCAAGAAATTGAGATTGGTCATGATATCATTCAGGTAACCAACCATAACG
CGCACCAAGACCAGTATCGTACTCCGGCAATTACCAACATGGTCAGTCTGGCGTTAGATTCAGTAGCACCTATTGACATGAACTGGTTCCAGGTTCCTGGTCGTGAGTTGTTCTTGAAGGTTGTTC
GTATTAATGGACAACCGGTTGGTTGTATCACACACTTACACACGGGACCAGGTCGTACCGTTAATGTTACTGGATTACTGTACACACGGAGTGATAATCACGGGAA
>xx12_212
TCGTATATTGAATGATGTTACTGTTGGACCTCACGGTGCTTTGTTCTATATTTATAATCATGAACCAACTGGGGTTGCAGGTTCTATCGTATTCACCCAACCAGAGCCCTGGAAACATTACGCTAC
CATTTATGTCGGACATAAGGATGAGTATGTTGATGTTAAACGCACCACCCCGTTTACTCGACCTGACATCATAAACAACGCTGTTATTGAAGCTGTAACAGAGTTCTTGAAGGATAAGGTAGAACA
GGAACATATGAAAGAGATGTATTCTGAAATTAACGAATACTTCCGTGTTCGACATGCTGCGTTTCTCCGGGTCATCTTCGATCTGAATAATAACTTCAAACTGTGAGATAACCATGTTAACTCGAT
CAAGTGCCAATCAACCATTACGGTTTAATGGAACTCCCTATCCTAATGAAGATAGGT
>xx12_222
CCAATATCAGGACCGAACGGATTACGTTCGTTCATGATCTTATTCATGTCGATCTTAGTCCCAGGTACCAGATCTACAGTACGGGCTTTACGAACGAACACAAAGTTATCAGTCAGTTCAAGAACG
ACAGAATTACCGACAGCCTTCACGAGACCAGGGCCGCTGGTTATCTTAGAACTGGTAATACCGATGTCCATATCTGGAATGATTGAAAGAAGCTCGTCAACCAGAATTGATGGAATGTCTGTAATA
GCCGGACCCATGCTCTTACCGATACAGATCAATACAGATTTATTACCTTCAGGGAACAGTGTGTCGAGTTTAACAAATGTTTCTTCAAGAGCAACCACCGTACCAATGTCAATATCGAGACCGCGT
GTCTTAGTTGTCGCCCATTTGCGATCTTTGTATACAACCCCAATATCAGC
>xx12_258
CTGTAATACAAAAGTAACTAAAGAGGTGATTGAAGAAGCCCAAGGTACCGCCCAAGCTATCTGCGACTACAAAAACAAATCACTGTAATTGATTTGATATAAAGGAAATTCGTTATGTTGGTAAA
CAAAGAAAACATCGTATCTTTTAGTCGTTCTGTCGAAACAGCGATCTCCCTGATGGGTGATAAGTTATTCCAAGATATTGGAGATAATCTCACCCTCATTAAAACCATTACTGATGGTCACCCCCT
TTACCGGTTGTATGATAAAGGTATCTCTGTTAAGGGCTATATCTGTGCCACGCCCGTAATGGTCTCTATTAGTGATTCCGTTGAGGCTATCATTGGTGGACTTATTGAAGATGATGTCGCTAAGAT
CGTATCAGATAT
>xx12_260
CTTCAAGACGGTCACGCGGGATACCAACAACAACCCAACCTTCATCAACACATGACAGAGTTAGGTCATAACACTCAGACAACCGCGATACCTCTTTATCAATACGTTCATCGACTGGAAGTGGTT
GGTAACGATAGGTCGCGATATTAACATGTGGATGAAGTGCGGTCATCGACACAACCATATCGACATATGCTTCATTGACATCCTCATCGTAATCTGTCTGATGAGGTAACAGAAGAAGGATGCCAG
TGACATTACTATCAATGGCTTGCATCAAGGAAATGCGTTTGTACTCTCCCAAATAAGGTTCCATATTAACCAAGAAAGTCCATTTAGGTTCCGCCAATATCAGTACCGAACGGATTAAGTTCGTTC
CATTCACTT
>xx12_85
CTAGAGGATACTTTAATAACGAAATTTTTGTTACGCGCAAGTATATTAGCGGCAATGATCCTCAGTCGGTTAAGGAAGAGATTGTCTACCACAACGAGTCGCCAGCATATCACACAAATGGCATTC
TAATTATCTCGGATAGTGTTCAGCACTTTCACGATCCAAAAACCTCTACTGACTACGCGGGTGTGACATTCACTACTGTTTCAAAGCTGAATGTTGGTAATCCCTCGGTTGGAGATATCGATACGT
TTACCATTGTATTCGATTCGGCGAATAACGCCACCCAGATATTAAGACGTTATGGGGAAAGCGATCCGCAAACCAACGGCAAGGGCTGGATGATCTCCTACAACGTTGGGGAAGAACCCCGACAAG
TGAGAGATATTCCTGAAGTGAGGAAGGTTATCGCGGGTTCGGACAAATAAACCCAATATATCTATAGCGTCGTATGCCGCATATGCTATACTATGAGCATATGCGGCATCTCAAACTATTTCTCAT
ATCCTCCGTCGGCGTATTTGGAGCCGTGTTGATATCGACACTCGCTATACCCGCCAACACAGTATACGCAGACGGGGTCACGATAAATGACATCGTCGCGCTCAAGGATCAATATTCGGTCAAAAA
CGTTCCGGGAAATTCAGCGGGTCCCGCATACGATATGCCATACTCGATCACCCTAAAAAGCCCGATATTTATAAACGGTGAACACACCTATGTTCTCGGTAACGCTAGTAGCACCAACACTGTGGG
TCTGAAATGCGGCGGCAATCAGCTGATGGCAACGTTGCTATTTAACAACGATTCTCTCAATGATCAGTTACGCGGAGGTAAAC
>xx12_94
CACTTAAAACAATACCCGGTCCATTCCAATTCCCAGAAGAGATCGGCGTAGTCCTTTTCTTTACGACCAGCTGAAAGTAGTACACCAGGAGCGATATCTTCCAGATGTTCTACTAGTACGTAAGGG
ACATTACCCATGGTACCATTGTTAGCGCTGGCTGACGTATAGATGTACTTAGTGTCATATGGACAATAAGCGATCATTGCGGTGATGGGTGTAACAACTGACTTAGGTGTGTACTCTTTCAATACA
ACGCCAAGCGTGAATTCCACCAACATGTTATTTCTCCGTTGATAAATAACGACGAGCTTGTTCAATAACTTCATTCATCATGAAGTCACCCTGCACATCATCAATGTTATTACGTACAGCTTTAAT
CGCATCACGGAACCCATTATACCAGTCTTCAGTTAAGAGGCGGGTAATCGTAATAATGGTATCACGACCATCAATGGTGTACACGTATTTCAGAACTACCGTCATATCGGTATGTTTGATATGGAT
AACGGTTCGAGTATTATCCAATAACTCGGCGGCCCCAGAAATGATAAGCTCATTACCCCATATCATAACCCGGGTTACTTCACTGAACTCAGACTCATCAACTTCGCGGTTGGTGATTTCATTCTG
ACGTTGTTCGATCAACCTAAACTTCTTAGAGTATTTGATCTTACAGTCACGCAGATAAGTACCGCCATTTTCATTGCGGGTATATTCGACCCAATCAATAAGAGGAAGGATATCGGAATGGAGTAA
CAAGTCCAGGGTATCTGGCAT
```

Table S1. Summary of eight contigs that cannot be annotated from virome analysis. Nucleotide sequences for these contigs are presented in fasta format.

BioProject #	Run #	# of reads mapped	Virome site	Subject	Ref.
PRJNA1110359	SRR29034544	5	Respiratory	Patients with respiratory illness	42
	SRR29034351	18	Respiratory		
PRJNA494633	SRR7963144	3	Respiratory		43
PRJNA686704	SRR13399360	1	Serum	Patients with arbovirus-like symptoms	SRA*
PRJNA906035	SRR25444810	1	Whole body	Tick	44
PRJNA666535	SRR16610293	7	Serum	Febrile Tanzanian children	45
	SRR16597575	7			
	SRR16610308	5			
	SRR16597517	4			
	SRR16597521	3			
	SRR16610045	2			
	SRR16597379	2			
	SRR16597516	1			
	SRR16597580	1			

Table S2. Screen of contig xx01_23 in virome sequencing data via read mapping. Initial screen of the NGS data from 118,731 bio-samples showed positive mapping in 14 bio-samples. Then, the NGS data from these fourteen bio-samples were subjected to read quality control to remove low quality reads and duplicates. The number of reads mapped resulted from the re-mapping with the data after read quality control. Asterisk indicates that patient phenotype infection was obtained from the National Center for Biotechnology Information Sequence Read Archive (SRA) portal. Ref., reference.
